# Mitral valve myxoma presenting with transient ischemic attack: a case report and review of the literature

**DOI:** 10.1186/s13256-018-1920-8

**Published:** 2018-12-08

**Authors:** Kendra J. Grubb, Vasa Jevremovic, Edgar G. Chedrawy

**Affiliations:** 1 Department of Cardiovascular and Thoracic Surgery, University of Louisville School of Medicine, Louisville, KY USA; 2 American Northwest University, Travnik, Bosnia and Herzegovina; 3 Cardiothoracic Surgery, Weiss Memorial Hospital, Chicago, IL USA; 4 Dalhousie University, Halifax, NS Canada

**Keywords:** Cardiac myxoma, Cardiac neoplasm, Mitral valve myxoma, Case report

## Abstract

**Background:**

Myxomas account for approximately half of all primary cardiac neoplasms. Most occur in the left atrium and only rarely are attached to the mitral valve, with just over 30 such cases reported in the literature. These neoplasms can manifest with a combination of obstruction of blood flow, systemic embolization, and constitutional symptoms.

**Case Description:**

We present a case of a 32-year-old African American man presenting at an emergency department with symptoms of a transient ischemic attack. Transesophageal echocardiography identified a mass originating from the posterior leaflet of the mitral valve. The mass was surgically resected and histologically classified as a myxoma. He remained asymptomatic during the course of 5-year surveillance.

**Conclusions:**

Few similar cases have been described in the literature. Here we present a review of the diagnosis and surgical management of this rare presentation for mitral valve myxoma.

## Introduction

Myxomas are rare stromal tumors of multipotential mesenchymal cell origin, capable of neural and endothelial differentiation [1]. As cardiac masses are predominantly secondary in nature, myxomas represent only a fraction of cardiac tumors, accounting for approximately half of primary cardiac neoplasia [2–5]. Arising predominantly from the atrial septum, the left atrial cavity is the site of 75% of myxomas [6]. Since 1871, there have been just over 30 reports of primary attachment to the mitral valve, including postmortem cases [2, 3, 5, 7]. There is a propensity for the tumor to originate from the atrial surface of the valve but attachment to either leaflet occurs in equal frequency. Transesophageal echocardiography (TEE) is the gold standard for non-invasive localization and diagnosis [8, 9]. Surgical treatment involves full thickness resection of the tumor with valve conservation or replacement and annuloplasty as necessary [10–13]. Long-term surveillance by TEE or transthoracic echocardiography (TTE) is necessary for early detection of recurrence [5].

## Case presentation

**Fig. 1:**
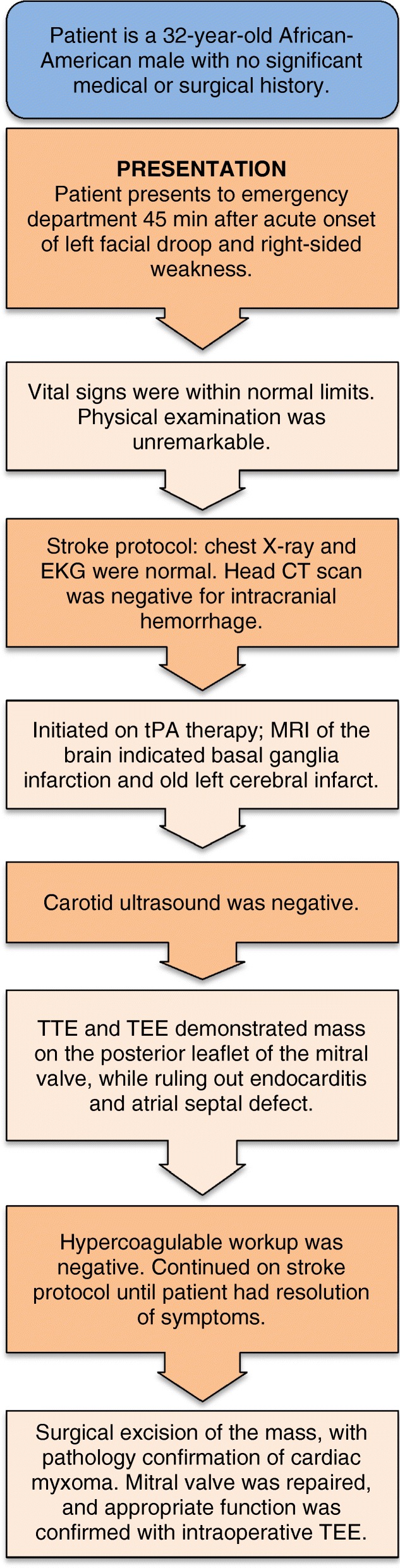
Myxoma case timeline. *CT* computed tomography, *EKG* electrocardiogram, *MRI* magnetic resonance imaging, *TEE* transesophageal echocardiogram, *tPA* tissue plasminogen activator, *TTE* transthoracic echocardiography

**Fig. 2:**
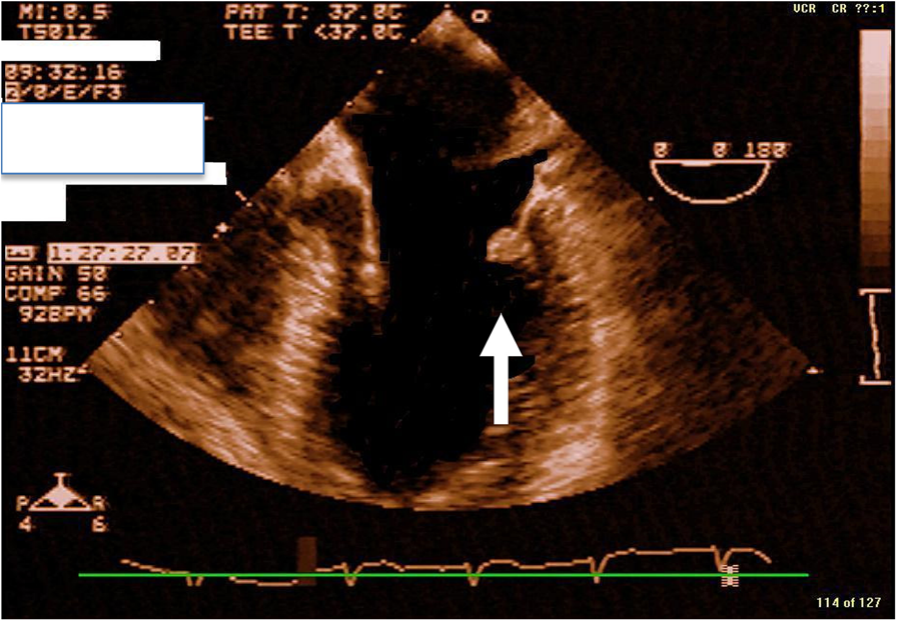
Transesophageal echocardiogram depicting mitral valve mass

A 32-year-old, previously healthy, African American man presented to an emergency department 45 minutes after the acute onset of left facial droop and right-sided weakness (Fig. 1). A thorough history confirmed an episode 1-week prior, during which he developed sudden onset of dizziness associated with nausea and vomiting that resolved within hours. He denied any past medical or surgical history and was taking no medications. He has no family history of tumors. In the emergency room, his vital signs were within normal limits. His physical examination was significant for a left facial droop and right hemiparesis. Auscultation of his chest revealed a regular rate and rhythm with no appreciable murmur. No additional significant findings were noted. Stroke protocol was initiated. A chest X-ray was normal and an electrocardiogram showed normal sinus rhythm. A head computed tomography (CT) scan was negative for signs of intracranial hemorrhage. He was subsequently started on tissue plasminogen activator (tPA) therapy. Magnetic resonance imaging (MRI) of his brain demonstrated a right basal ganglia infarct and an old left cerebral infarct. A carotid ultrasound was negative. TTE demonstrated a 1 cm by 1 cm mass on the posterior leaflet of the mitral valve with a moderate mitral regurgitation In addition, TTE revealed a questionable mass on the left coronary cusp of the aortic valve. These findings were confirmed with TEE (Fig. 2), which verified no sign of endocarditis and no atrial septal defect. A complete hypercoagulable workup was negative. Stroke protocol continued with the working diagnosis of cerebrovascular accident secondary to emboli from the mitral valve mass. Within 24 hours, he regained function of the right side of his body and had complete resolution of symptoms. He was diagnosed as having transient ischemic attack (TIA) and discussion was undertaken regarding surgical excision of his mitral valve mass.

### Operation

**Fig. 3:**
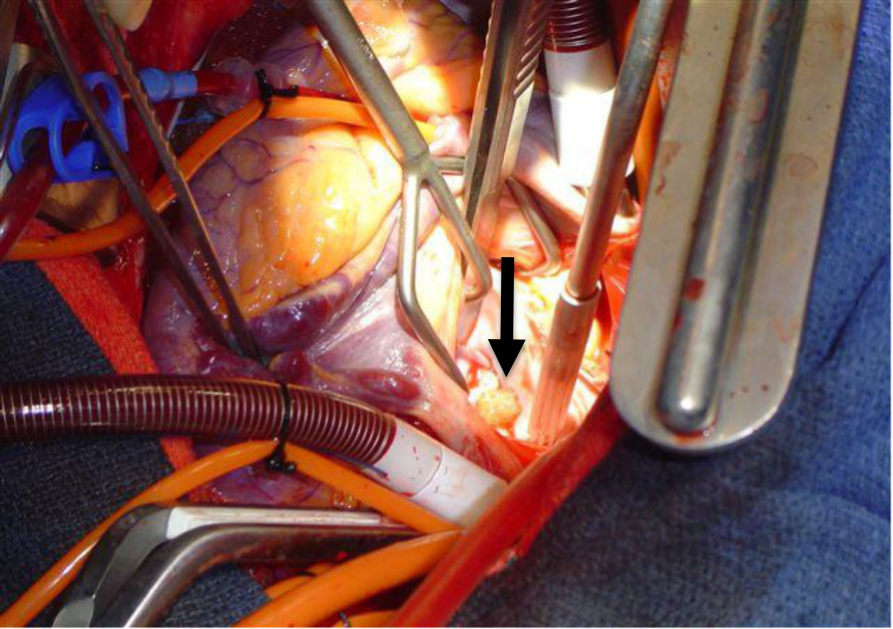
Intraoperative image: mitral valve myxoma. *Arrow* points to 2 × 3 cm pedunculated, soft, and polypoid mass arising from posterior leaflet of mitral valve

A median sternotomy was performed and cardiopulmonary bypass was employed via aortic and bicaval cannulation with full anticoagulation. His aorta was cross-clamped and his heart arrested with retrograde cardioplegia. The aortic valve was examined through an ascending aortotomy and all three valve leaflets appeared normal. A left atriotomy was made and the mass was easily identified on the posterior mitral valve leaflet adjacent to the mitral valve annulus (Fig. 3). The mass was excised and a frozen section confirmed globular myxoma cells with abundant eosinophilic cytoplasm consistent with myxoma. The valve leaflet was reconstructed with an autologous pericardium patch and the annulus was supported using a running DeVega-type suture. The valve appeared normal and was tested; no regurgitation was noted. His left atrium and aorta were closed. His aorta was unclamped, after aggressive venting and de-airing maneuvers, and his heart returned to normal sinus rhythm with successful weaning from cardiopulmonary bypass. Anticoagulation was reversed with protamine and his chest was closed after placement of drains and pacing wires. At the conclusion of the operation, TEE confirmed appropriate mitral valve function and normal aortic valve with no evidence of a mass and no regurgitation at either location.

## Discussion and Literature Review

Primary tumors of the heart are exceedingly rare; the incidence of primary tumors of the heart found at autopsy ranges from 0.0017 to 0.33% [14]. Myxomas account for approximately 50% of primary cardiac neoplasms with an overall incidence estimated at 0.5 per million population per year [15, 16]. They are considered to originate from multipotent mesenchymal cells that persist during septation of the heart [17]. Another proposed origin is cardiomyocyte progenitor cells or subendothelial vasoformative reserve cells in the fossa ovalis [17]. It is a rare finding for cardiac myxoma to arise from the mitral valve. Historically, the location has been 75% in the left atrium, 15 to 20% in the right atrium, and the remainder in the ventricle [3–5, 18]. Chakfe *et al.* performed a review in 1997 for all cases of mitral valve myxoma since they were first reported in 1871, including six postmortem and 21 clinical cases [19]. Subsequently, in 2001, Choi *et al.* updated the discussion and added an additional 10 cases to the body of knowledge [7]. In the 31 cases reviewed, mitral valve myxoma occurred slightly more frequently in females, and presented at a mean age of 37.6 ± 20.5 years. This is significantly younger than the age distribution for all presentations of myxoma [3, 4, 18]. This also differs from the average ratio of female to male presentation of left atrial myxoma in general, ranging from 2:1 to 3:1 [6].

### Presentation

The classic triad of obstructive symptoms, systemic embolization, and constitutional symptoms occur in conjunction infrequently. However, one of the three attributes may signify this entity on its own [6]. Obstructive symptoms of blood flow occur in 54 to 95% of patients in a valvular “ball-valve” mechanism [6]. This predominantly entails cardiac failure, accounting for 43% of cases in one series of studies, with dyspnea and lower extremity edema as predominant symptoms [6]. Systemic embolization occurs in 10 to 45% of patients with myxoma, with roughly two-thirds occurring in the central nervous system [6]. The literature documents cases of embolization to the extremities, aortic saddle, coronary arteries, kidneys, liver, spleen, and eye [3–5]. Constitutional symptoms occur in 90% of cases [3–6]. These non-specific markers of disease can include myalgia, arthralgia, muscle weakness, fatigue, fever, weight loss, anemia, elevated erythrocyte sedimentation rate, leukocytosis, and thrombocytopenia [3–6, 18, 20]. The presentation mimics many clinical scenarios such as syncope, collagen vascular disease, rheumatic heart disease, disseminated malignant disease, and infective endocarditis; thus, diagnosis is often made during a workup for these cardiac dysfunctions or disease processes [4, 21]. Cardiac myxoma typically presents with obstructive symptoms, but embolization of tumor or adherent clot occurs in 30 to 40% of patients with myxoma at any location [5, 22]. In addition, neurological symptoms due to embolism and auscultation abnormalities occur more frequently with patients of a young age [6]. Tumors arising from the mitral valve more often present with symptoms of embolization [7, 19]. The higher risk of embolization has been attributed to motion of the valve leaflets and the high pressure of the left ventricle [18, 19]. This may explain why patients present at younger ages and have few constitutional symptoms, as even small tumors can initiate embolic events.

### Diagnosis

Before the advent of angiocardiography in 1951, cardiac myxoma was only diagnosed at autopsy [6]. A thorough history and high index of suspicion are essential to making the diagnosis of mitral valve myxoma. Typically, the patient is young and may or may not have cardiac symptoms. In patients presenting with embolic symptoms, a complete workup for stroke or TIA must be undertaken. Messe and Jauch outlined the evaluation of TIA and necessary workup: routine blood tests (glucose, serum chemistry, complete blood count, urine analysis, and coagulation profile), tests for hypercoagulability, a cardiac evaluation (cardiac enzymes, electrocardiography, cardiac monitoring, and consideration of echocardiography), cerebrovascular imaging (CT or MRI with angiography), and vascular imaging of the carotid arteries [23]. In the case of a mitral valve myxoma, routine laboratory tests and initial cardiac evaluation are usually negative. A TTE is then performed and, if a mass is identified, a TEE is undertaken to further delineate the anatomical details of the site of attachment [9, 18]. TEE is the gold standard for non-invasive diagnosis and localization [8, 9, 24, 25]. Very small lesions, less than 0.5 cm by 0.5 cm are not typically visualized by TTE and therefore TEE examination in all patients with unexplained TIA or stroke must be employed [19, 26]. A review by Borges *et al.* reported that three-dimensional TEE can provide exact spatial information regarding the shape and surface of the mass, clarify the involvement of the mitral valve, and identify any obstruction of the mitral valve annulus or laceration of the mitral valve leaflets [8]. They further suggested that it is possible to simulate intraoperative visualization of cardiac structures and surgical anatomy for operative planning. Additional details necessary for surgical planning can be gained from MRI or CT of the chest [5, 27]. On macroscopic examination, cardiac myxoma may assume a polypoid or papillary form [17]. Histopathologic diagnosis is reliant on identification of myxoma cells, which are arranged singly, in small clusters, or capillary-like channels in a myxoid stroma [17]. These cells can be spindle, stellate, or polygonal, with round or oval nuclei and inconspicuous nucleoli, rarely with mitoses [17]. On immunohistological examination, cardiac myxomas are diffusely expressive for vimentin with focal expression of CD34, CD68, and SMA [17].

### Treatment

Prompt surgical excision of a mitral valve myxoma must be undertaken as the patient is at risk for additional embolic events. Sandrasagra *et al.* reported the first surgical excision of a myxoma of the mitral valve in 1979 [28]. The approach included a radical excision of the mass and valve replacement. Management has changed little in the interim, as treatment today must include complete resection of the mass with tumor-free margins followed by repair or replacement of the valve and annuloplasty when necessary [11]. There is no consensus as to the exact approach to these ends, making each course of management situational. Jones *et al.* reviewed a 30-year experience, and in 1995 presented the largest series to date of operative approaches to cardiac myxoma [10]. Their review included all presentations of cardiac myxomas and supported a biatrial approach, in order to fully inspect all intracardiac surfaces for synchronous lesions and provide adequate exposure for en bloc resection. However, in the modern era, thorough preoperative imaging and the addition of intraoperative TEE provide sufficient data to exclude additional lesions. Furthermore, there have been no reports of synchronous right heart lesions in the review of all cases of mitral valve myxoma [7, 19]. Regarding management of the mitral valve post resection, there is no current standard of care. Most valves are reconstructed, leaving valve replacement for cases of large tumors with significant defects, ventricular side tumors, and myxomas involving both leaflets [7, 29–31].

### Follow up

Patients must continue to be followed with serial examinations for early detection of recurrent disease. There is no standard protocol for surveillance; however, it has been our practice to perform an annual physical examination and complete a TEE at that time. The presented patient has been followed for 5 years and has had no sign of recurrence and had normal functioning valves on serial TEE examinations.

## Conclusion

Although mitral valve myxoma is a rare clinical entity, the diagnosis should be considered in all young patients presenting with symptoms of stroke. These patients should undergo echocardiography early and prompt surgical resection must be undertaken once the diagnosis of mitral valve myxoma has been made. We present this review for further discussion of this rare cardiac neoplasm.

## Abbreviations

CT: Computed tomography
MRI: Magnetic resonance imaging
TEE: Transesophageal echocardiography
TIA: Transient ischemic attack
tPA: Tissue plasminogen activator
TTE: Transthoracic echocardiography
